# UniProt: the Universal Protein Knowledgebase in 2025

**DOI:** 10.1093/nar/gkae1010

**Published:** 2024-11-18

**Authors:** The UniProt Consortium, Alex Bateman, Maria-Jesus Martin, Sandra Orchard, Michele Magrane, Aduragbemi Adesina, Shadab Ahmad, Emily H Bowler-Barnett, Hema Bye-A-Jee, David Carpentier, Paul Denny, Jun Fan, Penelope Garmiri, Leonardo Jose da Costa Gonzales, Abdulrahman Hussein, Alexandr Ignatchenko, Giuseppe Insana, Rizwan Ishtiaq, Vishal Joshi, Dushyanth Jyothi, Swaathi Kandasaamy, Antonia Lock, Aurelien Luciani, Jie Luo, Yvonne Lussi, Juan Sebastian Martinez Marin, Pedro Raposo, Daniel L Rice, Rafael Santos, Elena Speretta, James Stephenson, Prabhat Totoo, Nidhi Tyagi, Nadya Urakova, Preethi Vasudev, Kate Warner, Supun Wijerathne, Conny Wing-Heng Yu, Rossana Zaru, Alan J Bridge, Lucila Aimo, Ghislaine Argoud-Puy, Andrea H Auchincloss, Kristian B Axelsen, Parit Bansal, Delphine Baratin, Teresa M Batista Neto, Marie-Claude Blatter, Jerven T Bolleman, Emmanuel Boutet, Lionel Breuza, Blanca Cabrera Gil, Cristina Casals-Casas, Kamal Chikh Echioukh, Elisabeth Coudert, Beatrice Cuche, Edouard de Castro, Anne Estreicher, Maria L Famiglietti, Marc Feuermann, Elisabeth Gasteiger, Pascale Gaudet, Sebastien Gehant, Vivienne Gerritsen, Arnaud Gos, Nadine Gruaz, Chantal Hulo, Nevila Hyka-Nouspikel, Florence Jungo, Arnaud Kerhornou, Philippe Le Mercier, Damien Lieberherr, Patrick Masson, Anne Morgat, Salvo Paesano, Ivo Pedruzzi, Sandrine Pilbout, Lucille Pourcel, Sylvain Poux, Monica Pozzato, Manuela Pruess, Nicole Redaschi, Catherine Rivoire, Christian J A Sigrist, Karin Sonesson, Shyamala Sundaram, Anastasia Sveshnikova, Cathy H Wu, Cecilia N Arighi, Chuming Chen, Yongxing Chen, Hongzhan Huang, Kati Laiho, Minna Lehvaslaiho, Peter McGarvey, Darren A Natale, Karen Ross, C R Vinayaka, Yuqi Wang, Jian Zhang

**Affiliations:** European Molecular Biology Laboratory, European Bioinformatics Institute (EMBL-EBI), Wellcome Genome Campus, Hinxton CB10 1SD, UK; Protein Information Resource, Georgetown University Medical Center, 2115 Wisconsin Ave NW, G1 level, Suite 040A, Washington, DC 20007, USA; Protein Information Resource, University of Delaware, Ammon-Pinizzotto Biopharmaceutical Innovation Building, Suite 147B, 590 Avenue 1743, Newark, DE 19713, USA; SIB Swiss Institute of Bioinformatics, Centre Medical Universitaire, 1 rue Michel Servet, CH-1211, Geneva 4, Switzerland

## Abstract

The aim of the UniProt Knowledgebase (UniProtKB; https://www.uniprot.org/) is to provide users with a comprehensive, high-quality and freely accessible set of protein sequences annotated with functional information. In this publication, we describe ongoing changes to our production pipeline to limit the sequences available in UniProtKB to high-quality, non-redundant reference proteomes. We continue to manually curate the scientific literature to add the latest functional data and use machine learning techniques. We also encourage community curation to ensure key publications are not missed. We provide an update on the automatic annotation methods used by UniProtKB to predict information for unreviewed entries describing unstudied proteins. Finally, updates to the UniProt website are described, including a new tab linking protein to genomic information. In recognition of its value to the scientific community, the UniProt database has been awarded Global Core Biodata Resource status.

## Graphical Abstract

**Graphical Abstract:**
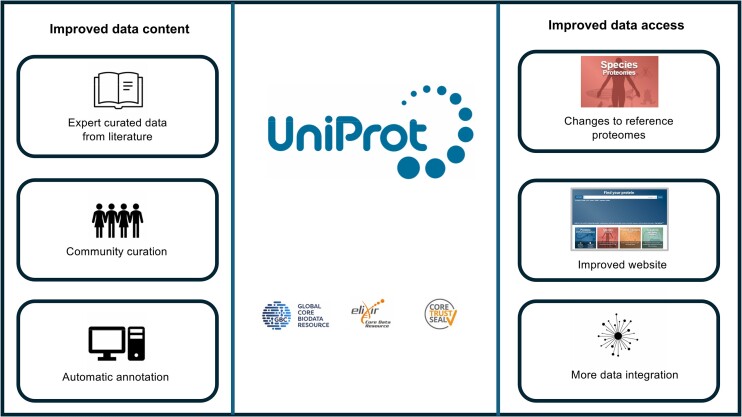

## Introduction

The UniProt suite of databases (https://www.uniprot.org/) serves as a leading global data resource for protein sequence and functional information (1). The long-standing critical role of the data resource in underpinning molecular biology was recognized when UniProt became one of the first databases awarded Global Core Biodata Resource status (https://globalbiodata.org) in December 2022. The central UniProt Knowledgebase (UniProtKB) is a curated resource. It is comprised of both a reviewed set of protein entries (UniProtKB/Swiss-Prot), where each record contains a summary of the experimentally verified, or computationally predicted, functional information, added and evaluated by an expert biocurator, and the unreviewed set (UniProtKB/TrEMBL). In the latter, entries are computationally annotated by automated systems. The UniRef databases cluster sequence sets at various levels of sequence identity and the UniProt Archive (UniParc) delivers a complete set of known unique sequences, including historical obsolete sequences. If a UniParc entry sequence is not included in UniProtKB, the reason for the exclusion of that sequence is provided (e.g. pseudo-gene). In addition, each UniParc record contains a reference to its source database(s) with accession and version numbers, tagged with its status in that database, indicating if the sequence still exists or has been deleted in the source database.

Historically, UniProtKB contained all protein sequences deposited by the research community in any International Nucleotide Sequence Database Collaboration (INSDC) member resource (2) in addition to proteomes derived from selected genomes annotated by either Ensembl (3) or RefSeq (4). In the future, as the rate of whole genome sequencing increases and global projects aim to sequence across the whole biodiversity of species and, in some cases, multiple strains, sexes or variants of a single species, this will become increasingly difficult to maintain and for our users to navigate and comprehend. We describe here our response to managing the increased number of genomes now available in the public domain, and their effect on our production pipelines. In addition, we outline our work to improve the efficiency of information retrieval and summarization from the scientific literature and also from collaborating repositories, and the enhanced representation of these data in UniProtKB records.

## Progress and new developments

### Managing the sequence space

In May 2024, the INSDC contained 2.4 billion nucleotide sequences, many with associated translations to protein sequences made by the submitter, which form the basis of gene calls by groups such as Ensembl or RefSeq. Most of these sequences are highly redundant, i.e. identical or near identical in sequence content derived from a single organism which has been sequenced many times or from the sequencing of very closely related species. UniProtKB would not supply an optimal service to our users if we included all of the sequences available from INSDC or downstream annotation sources. Redundant sequences do not add significantly to information content, and their presence both slows down and compromises the output of services such as BLAST (5) and are costly to process and perform repetitive computation upon. UniProt release 2024_04 contains approximately 246 million sequence records in UniProtKB. This represents a relatively conservative increase in sequence number over the last 2 years (6), reflecting measures already put in place to limit the volume of data entering the Knowledgebase. As data are automatically imported from the INSDC, we filter to exclude redundant copies of proteomes from the same organism (identified by taxonomic identifier) and increasingly remove clinical isolates where, again, multiple copies of the same genome are sequenced, such as happened during the recent pandemic. Imports from Ensembl, RefSeq and other genome annotation platforms such as WormBase ParaSite (7) are still largely manually screened as import candidates. We continue to assess the quality of the remaining proteomes, using BUSCO (8) and the complete proteome detector (9). We then implement additional quality checks based on criteria developed by RefSeq, supplemented by further internally created checks. Only the highest quality, nonredundant proteomes remain to be submitted to our process for automatically selecting reference proteomes, i.e. a representative of each group of a cluster of proteomes grouped by overall sequence similarity. We additionally manually select and maintain a number of key reference proteomes, thus ensuring that those derived from model organisms or selected by the community as important for a particular area of study are tagged and remain the reference for their cluster group. To ensure stability in proteome selection, the pipeline is weighted to ensure that once selected a proteome remains representative for its group unless a related proteome which is significantly improved in quality (in terms of genome standard, coverage and annotation) becomes part of the cluster. This is then evaluated as a replacement for the existing representative proteome.

It is our intention in 2025 to limit the unreviewed sequences in UniProtKB/TrEMBL to only those derived from reference proteomes, unless there is significant additional functional information associated with an entry, for example a 3D structure, or there is a request from the community that a set of entries be included. This will initially result in a drop in the number of unreviewed entries in UniProtKB, which will then increase as biodiverse sequencing projects lead to the creation of novel proteome clusters. Redundant proteomes will continue to be available for search and download in UniProt through UniParc and we will work to improve user access to this resource. It should be noted that the selection of viral reference proteomes is a separate process. The small size of their proteome means that our redundancy pipeline is ineffective so viral reference proteomes are selected manually, in collaboration with the International Committee on Taxonomy of Viruses (10).

### Expert curation

The extraction and evaluation of information relating to protein sequence, structure and function from the scientific literature and the addition of these data to the relevant UniProtKB/Swiss-Prot reviewed records is of central importance to the operation of the UniProt data resource. Expert curators target key publications and create or update records pertaining to human and model organism biology, including enzymology, host–pathogen interactions and many other areas critical to the studies of biodiversity and human health and disease. Over the last few years, a particular effort has been made to retrofit enzyme and transporter annotation using the Rhea knowledgebase of biochemical reactions, which uses the ChEBI ontology to represent reactants (11,12). At the time of writing, UniProtKB includes annotations to 12,501 Rhea reactions, which are linked to 28,259 857 UniProtKB protein sequence records, including 231,709 reviewed protein sequence records in UniProtKB/Swiss-Prot. We also actively track publications describing new enzymatic reactions or orphan reactions. One example of this is the identification of the flavin reductase BLVRB (https://www.uniprot.org/uniprotkb/P30043/entry) as a moonlighting enzyme. Previously known to be involved in fetal heme catabolism in the liver, a recent publication showed that BLVRB also catalyzes the S-nitrosylation of cysteine residues of specific target proteins, including INSR and IRS1 thus inhibiting insulin signaling (13). Whilst the protein S-nitrosylation modification had been known for many years to act as a key mediator of signal transduction, and is described in more than 60 human entries in UniProt, (https://www.uniprot.org/uniprotkb?query=(keyword:KW-0702) the enzymes that mediate this modification were previously largely unknown. We curated the publication in UniProtKB/Swiss-Prot and provided functional information in the form of human readable text summaries and structured vocabularies, such as the Gene Ontology (GO) (14), Rhea or ChEBI (15). We created new reactions to describe the multistep transnitrosylation reactions in the BLVRB entry and described protein S-nitrosylation sites and their effect on target entries [HMOX2 (P30519), INSR (P06213) and IRS1 (P35568)]. The use of the ChEBI ontology to describe chemical structures for both enzyme reactions and post-translational modifications (PTMs) provides a direct link between enzymes and their targets, thereby increasing the interoperability of UniProt. UniProt curators are also highly involved in the curation of biological systems using Gene Ontology–Causal Activity Model(s) [GO-CAM(s)] (15,16) in order to describe the flow between molecular functions. We curated a GO-CAM to describe the BKVRB catalyzed reactions (https://www.alliancegenome.org/gene/HGNC:1063#function—go-annotations) and are currently working on the import and display of GO-CAM models on the UniProt website.

**Figure 1.**
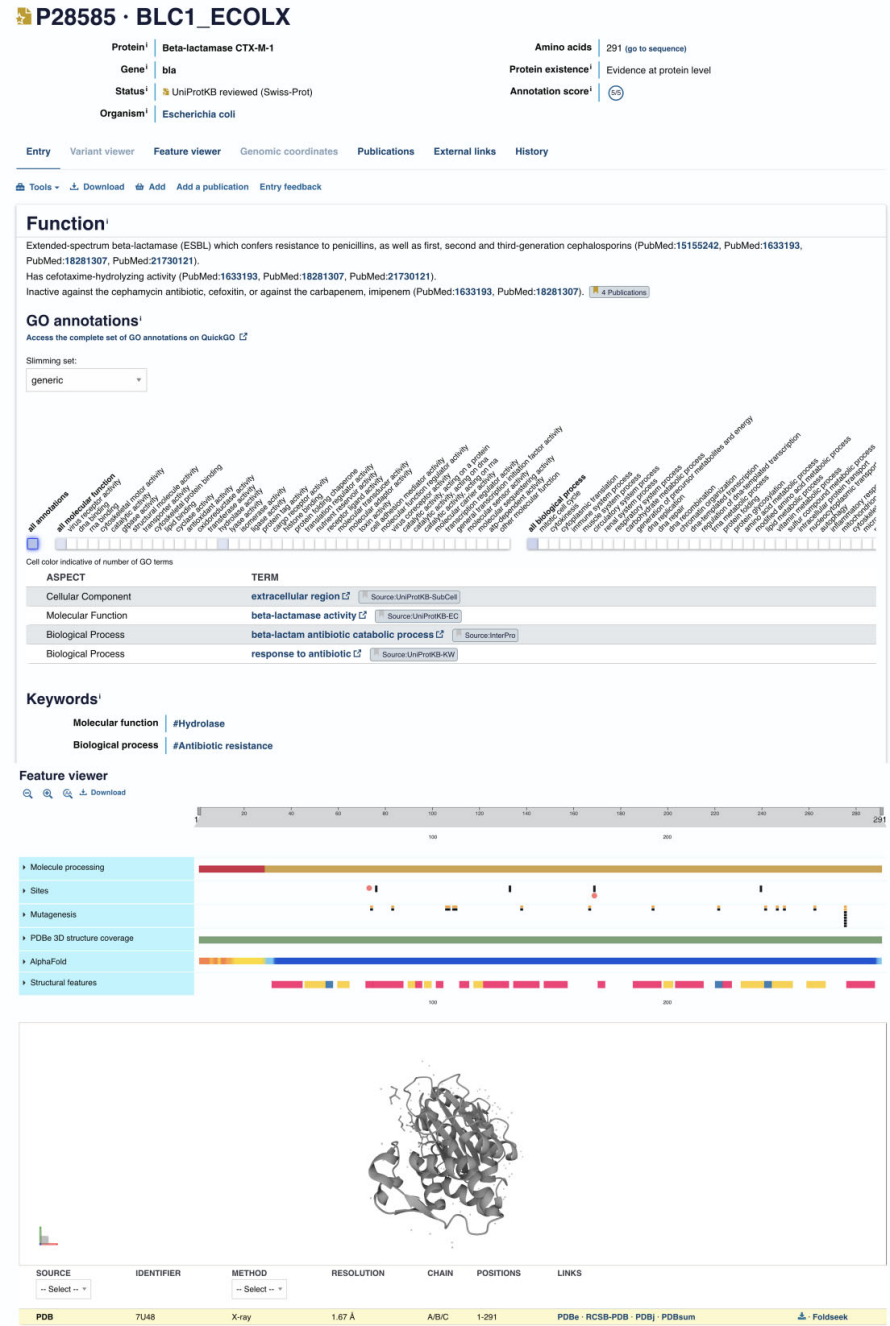
The annotation of an extended-spectrum beta-lactamase (P28585) which confers resistance to penicillins.

In a separate project, we have been proactively collaborating with research teams working in the area of antimicrobial drug resistance (AMR), focusing on mechanisms of drug resistance developed by the World Health Organization Class I target ‘ESKAPE’ organisms (*Escherichia coli, Staphylococcus aureus, Klebsiella pneumoniae, Acinetobacter baumanii, Pseudomonas aeruginosa* and *Enterobacteriaceae*). We are identifying and updating key proteins playing direct roles in AMR, such as beta-lactamases (Figure 1), carbapenemases, efflux pumps and ABC transporters are currently being targeted for annotation with efforts expanding to include members of two-component signalling systems, quorum sensing proteins and those involved in biofilm formation in the near future.

As the volume and diversity of the scientific literature increases, we are increasingly turning to machine learning (ML) techniques to assist the curation process with selection of key papers and data extraction. Existing ML frameworks such as LitSuggest (17) are used by UniProt curators to identify papers on subjects such as amino acid variants linked to human disease, enzymatic reactions and protein complexes. UniProt members have collaborated with the PubMed team at NCBI on the development of a new training and benchmarking dataset, EnzChemRed, to support the development of Natural Language Processing models to assist enzyme curation (18,19). EnzChemRED can boost the ability of BERT (Bidirectional Encoder Representations from Transformers)-based and GPT (Generative pre-trained transformer) models to extract novel enzyme–substrate relationships from the literature.

The potential use of large-language models (LLMs) to generate text directly from the scientific literature is currently being actively evaluated by UniProt curators. We are aiming to achieve an LLM-generated ‘first-pass’ summarization of relevant publications to add value to unreviewed entries and supplement existing information in UniProtKB/Swiss-Prot records. A number of pilot projects are currently underway evaluating the precision and recall of existing models and also the potential of LLMs to act as a curator-assist tool, increasing the throughput of publications which can be evaluated in the expert curation process.

### Community curation

**Figure 2.**
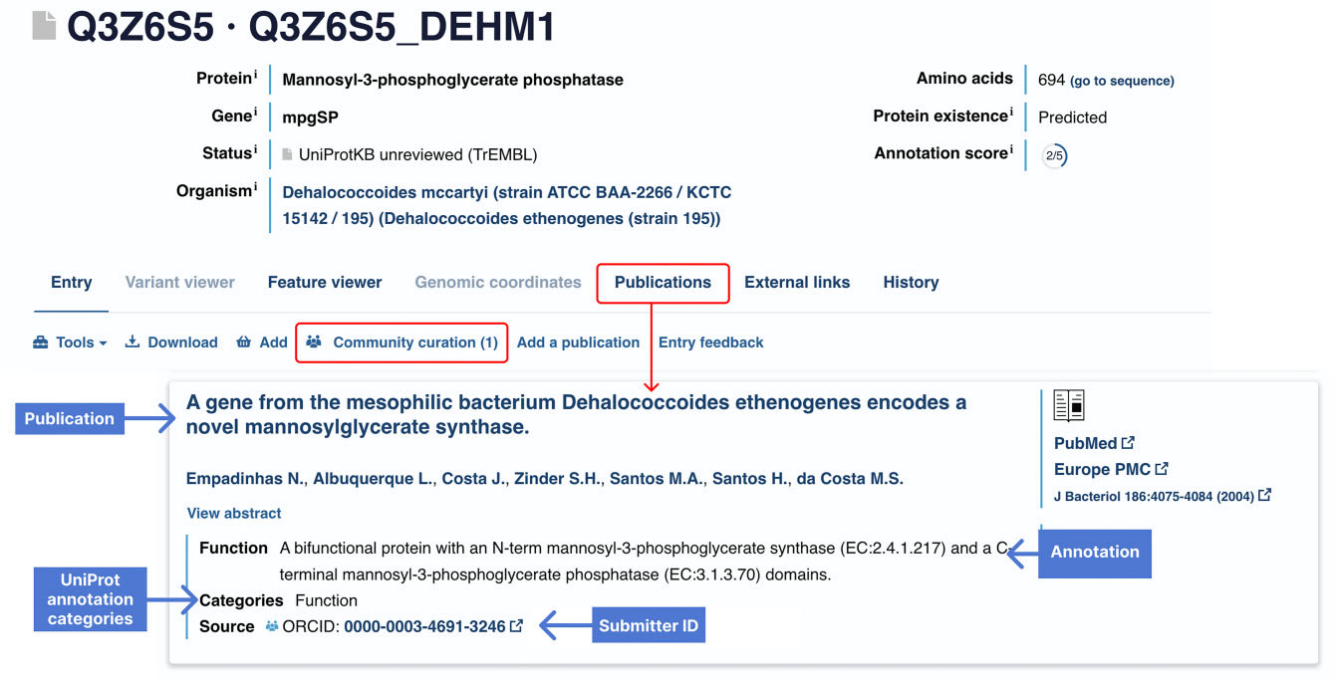
Community submitted information is now added to relevant UniProtKB entries.

As previously described (6), we continue to reach out to the broader scientific community, asking researchers to alert us to entries requiring an update and novel publications carrying key pieces of information. Submitters are directed to a simple form which enables them to enter information from a publication and select appropriate annotation categories to which information can be added. A ‘Batch Submission’ option enables upload of a collection of publications and annotations for one or more entries. As of release 2024_04, there have been 3967 submissions enabling update of 3504 proteins with 1744 publications (https://community.uniprot.org/bbsub/STATS.html). Each of these submissions has been reviewed by a UniProt curator and submitted information is now visible on the UniProtKB webpages for each updated entry (Figure 2). Contributors are identified by their ORCID and a link to their ORCID landing page validates their expertise. We are now looking to actively solicit community submissions by identifying relevant papers through text-mining and reaching out to the authors to help evaluate the text-mining results and suggest further annotations which can be extracted from the publications. We are also exploring the use of LLMs to generate draft annotations that can be sent to paper authors for review.

## Automatic annotation and ML

Adding functional information to the body of unreviewed entries in UniProtKB/TrEMBL remains an important task. These records are enriched with functional information by annotation transfer systems, which use the protein classification tool InterPro to group sequences at superfamily, family and subfamily levels, and to predict the occurrence of functional domains and important sites. The rule-based computational annotation UniRule system (20) uses these groupings to transfer experimentally verified annotations onto unstudied proteins, adding properties, such as protein name, functional annotation, catalytic activity, pathway, GO terms and subcellular location. We continue to expand this rule set, most recently with rules created by them based on NCBIfam signatures (21) evaluated and added to the set.

The Association-Rule-Based Annotator system for automatic classification and annotation of UniProtKB proteins (9) has been further developed, most recently through the incorporation of novel PANTHER signatures (22). This immediately generated 9141 new rules and 119,579,654 new predictions for over 20 million new protein sequences. We are additionally collaborating with Google Research who have developed ProtNLM, an LLM that accurately predicts descriptions of protein function directly from a protein’s amino acid sequence (23). Initially, a single model was pre-trained, using the T5 framework, across the UniRef50 2018_03 dataset of ∼30 million diverse unlabelled protein sequences, then fine-tuned using labelled protein domain sequences from Pfam (24). This has since been extended to encompass the output of multiple models. To date, ProtNLM has been used to annotate more than 28 million proteins (2024_04) previously labelled as ‘uncharacterized’ with a functionally relevant name and we are actively exploring options with Google to extend this annotation to additional fields.

We continue to make our data more accessible to ML algorithms, to act as both training and test sets. We now make raw embeddings (per-protein and per-residue) available for UniProtKB/Swiss-Prot and some reference proteomes of model organisms. These have been generated using the bio_embeddings tool (https://github.com/sacdallago/bio_embeddings), prottrans_t5_xl_u5 model0, and can be retrieved from our Downloads page. Per-protein embeddings for UniProtKB can also be retrieved from the UniProtKB search results download function. We have also reached out to researchers to help us with our efforts to develop novel algorithms for use in the UniProtKB production pipeline and have led/participated in a number of community challenges. The UniProt metal-binding challenge invited the ML community to create computational methods to predict metal-binding sites across the whole of UniProtKB. This problem was chosen as currently ∼17% of curated proteins have annotated metal-binding site residues whereas only 3% of unreviewed entries have these predicted, giving a large search space against which successful predictions could potentially be made. We provided both a training set and a test set of 1 million proteins. Unfortunately, although several groups participated in this challenge, all predictions contained a high number of false positives. More successful was our participation in a CAFA (Critical Assessment of Functional Annotation) challenge on the Kaggle platform, in which participants were asked to predict the function of a set of proteins, as indicated by the use of GO terms (www.kaggle.com/competitions/cafa-5-protein-function-prediction/). UniProt curators selected and contributed to the annotation of a training set of annotated proteins and also provided a curated test set to assist in the evaluation stage. The number of entrants was significantly higher, and a good success rate was achieved, with the possibility of embedding some of the successful code into future UniProt pipeline development.

## Data integration

UniProtKB works with many other groups to access and integrate large-scale datasets, mapping these data onto the appropriate protein sequence records and displaying the mappings via the ProtVista Feature Viewer (25). All data shown are downloadable via File Transfer Protocol and Application Programming Interfaces (APIs). We have worked to improve our support for the mass spectrometry (MS) community (26). The existing UniProt MS peptide identification pipeline by which MS-based proteomic data deposited into proteomeXchange databases (27,28) is reprocessed, scored and used to verify the existence of each protein is being upgraded to meet the stringency of the HUPO Human Proteome Project (HPP) 3.0 guidelines (29). UniProtKB now actively supports the HUPO HPP which aims to provide MS evidence for the existence of each protein in the human proteome (Omenn *et al.* 2024, in preparation). All proteins identified by the qualifying number of unique peptides of a set minimum length are annotated with the keyword ‘proteomics identification’, and the protein existence level is set to ‘experimental evidence at protein level’.

We have additionally collaborated with PRIDE (29), PeptideAtlas (27,28) and the University of Liverpool to enable the integration of PTMs from these resources into UniProtKB records (30). Filtering and reanalysis ensure that only high-quality datasets are imported and reanalysed, with these datasets stored in PRIDE attributed a unique ID (PXD), thereby facilitating user access, traceability and reusability. Modified sites are assigned a confidence score based on their false localization rate across multiple datasets to reflect the strength of evidence available. Data are integrated into UniProtKB and visualized in the ProtVista Feature Viewer both in the PTM/processing section of the protein entry page and in the Feature Viewer in site-centric format. Modified peptide data are visualized in the proteomics section of the Feature Viewer, and both site-centric and peptide-centric data are accessible via the proteins API. At the time of writing, rice (*Oryza sativa subsp. Japonica)* and *Plasmodium falciparum* proteomic PTM data are available with *Saccharomyces cerevisiae*, mouse (*Mus musculus*) and human (*Homo sapiens*) data currently being processed.

## Website

**Figure 3.**
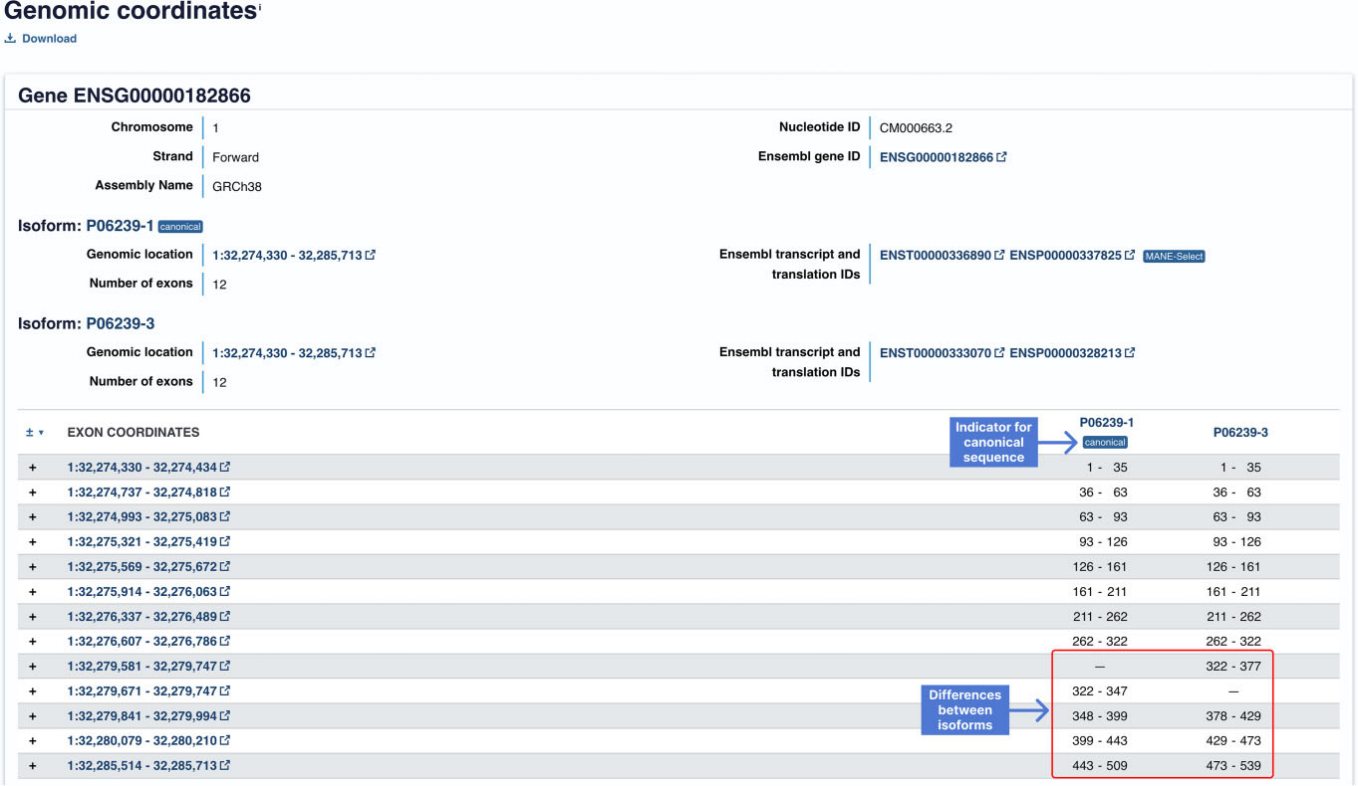
The Genomics Tab view for the human Tyrosine-protein kinase Lck protein (P06239).

We continue to develop the UniProt website with an emphasis on providing the user with an effective search, easy navigation and excellent responsiveness whilst further improving the tools dashboard and the programmatic access interface (API). The website has been built using a modular approach, separating the front end (web user interface) from the back end (API). We have added additional views to the website, first moving the variant view from its previous position embedded in the disease and variant section of the website to a separate tab to increase the overall speed of website loading. Second, we have created a genomics tab (Figure 3) which enables the user to link protein data to the corresponding genomic coordinates. Genomic coordinates of UniProtKB proteins are imported from the corresponding nucleotide sequence data in the INSDC. For those imported from Ensembl, Ensembl Genome, RefSeq and WormBase ParaSite, the genomic coordinates are also accessed from those resources. For each UniProtKB entry, representing the product(s) of one gene, details of the genomic assembly, chromosomal location of the gene and strand are given. Each protein isoform is listed with its corresponding genomic location, the total number and the genomic coordinates of the exons creating each isoform and the corresponding ranges of encoded amino acids.

## Visualizing proteins in the context of protein complexes

**Figure 4.**
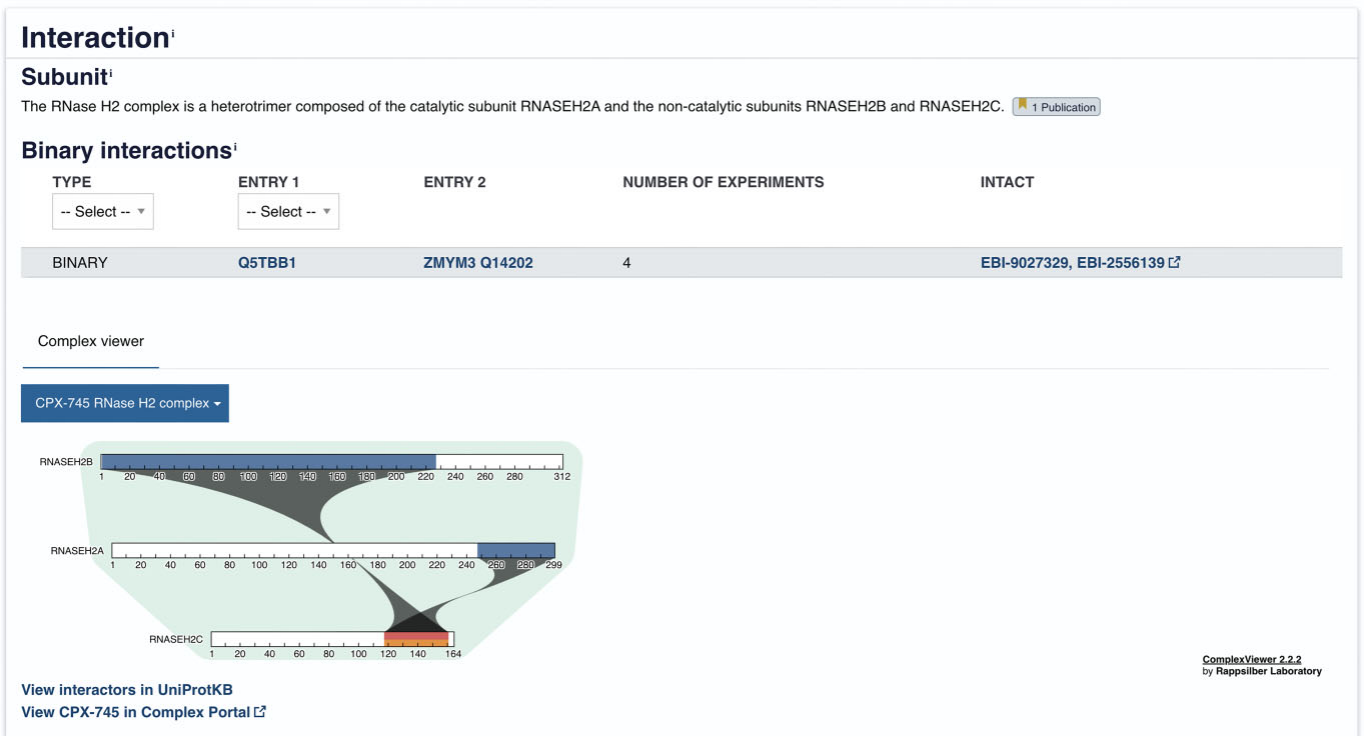
Visualization of the RNase H2 complex as seen in the UniProt entry for the Ribonuclease H2 subunit A protein (O75792).

Many proteins are functionally operative only as a member of a protein complex, a set of polypeptide chains, that interact with each other and/or with a nucleic acid. Mutations in genes encoding different proteins within the same complex cause similar diseases (locus heterogeneity). In order to present the user with a representation of a protein in its functional environment, we have collaborated with the Complex Portal (31) to provide structured, machine-readable descriptions of complexes using UniProtKB identifiers and a range of shared reference ontologies, such as the GO and ChEBI. To date, a total of almost 5000 manually curated complexes have been released with UniProtKB curators contributing significantly to that number. Complex Portal entries are cross-referenced in UniProtKB and we have added the ComplexViewer (32) to the UniProt website. This provides a dynamic, interactive, responsive 2D visualization of each complex, enabling users to see its topology and stoichiometry (when known) and zoom in on binding regions down to the residue level (Figure 4). Interactive links enable users to easily move between the UniProtKB entries describing complex components or to learn more about the full assembly in the Complex Portal.

## Conclusion

The UniProt database will undergo some fundamental changes over the next 1–2 years, as we adapt our data processing pipelines to address the increase in the amount of whole genome sequencing data which is now being produced on an almost industrial scale. At the same time, we wish to ensure our users enjoy the same quality of service we have offered them over the previous decades. We aim to present a reference proteome for each taxonomic grouping to the research community. Whilst we will continue to evaluate the scientific literature and summarize key information on proteins and their functions, new ML tools and language models mean we are approaching how we do so in a very different way. However, we will ensure that we maintain the high-quality, expert curation for which UniProtKB is known. We additionally encourage community contributions and now make those visible directly on the webpage of the relevant entries. We will continue to develop our methods for the computational annotation of unreviewed proteins and will increasingly work with the ML community through collaborations and challenges to enable this.

UniProtKB acts as a central hub of information integrating data from many external resources. We supply a number of APIs enabling users to access UniProt data or incorporate this into their own resources (www.uniprot.org/help/programmatic_access). We proactively collaborate with other data producers and data (re)users and are always looking for opportunities to extend such interactions. We greatly value the feedback and annotation updates from our user community. Please send your comments and suggestions via the contact link on the UniProt website (https://www.uniprot.org/contact).

## Data Availability

UniProt releases are published every 8 weeks. We provide customizable views and downloads in a range of formats via the website, and file sets at the FTP site (www.uniprot.org/downloads), and supply users with a number of different options for computational access to the data (www.uniprot.org/help/programmatic_access). These include the website RESTful Application Programming Interface (API), stable URLs that can be bookmarked, linked and reused, the SPARQL API that allows users to perform complex queries across all UniProt data and also other resources that provide a SPARQL endpoint and a Java API.

## Appendix

### The UniProt Consortium

Alex Bateman, Maria-Jesus Martin, Sandra Orchard, Michele Magrane, Aduragbemi Adesina, Shadab Ahmad, Emily H. Bowler-Barnett, Hema Bye-A-Jee, David Carpentier, Paul Denny, Jun Fan, Penelope Garmiri, Leonardo Jose da Costa Gonzales, Abdulrahman Hussein, Alexandr Ignatchenko, Giuseppe Insana, Rizwan Ishtiaq, Vishal Joshi, Dushyanth Jyothi, Swaathi Kandasaamy, Antonia Lock, Aurelien Luciani, Jie Luo, Yvonne Lussi, Juan Sebastian Martinez Marin, Pedro Raposo, Daniel L. Rice, Rafael Santos, Elena Speretta, James Stephenson, Prabhat Totoo, Nidhi Tyagi, Nadya Urakova, Preethi Vasudev, Kate Warner, Supun Wijerathne, Conny Wing-Heng Yu and Rossana Zaru at the EMBL-European Bioinformatics Institute; Alan J. Bridge, Lucila Aimo, Ghislaine Argoud-Puy, Andrea H. Auchincloss, Kristian B. Axelsen, Parit Bansal, Delphine Baratin, Teresa M. Batista Neto, Marie-Claude Blatter, Jerven T. Bolleman, Emmanuel Boutet, Lionel Breuza, Blanca Cabrera Gil, Cristina Casals-Casas, Kamal Chikh Echioukh, Elisabeth Coudert, Beatrice Cuche, Edouard de Castro, Anne Estreicher, Maria L. Famiglietti, Marc Feuermann, Elisabeth Gasteiger, Pascale Gaudet, Sebastien Gehant, Vivienne Gerritsen, Arnaud Gos, Nadine Gruaz, Chantal Hulo, Nevila Hyka-Nouspikel, Florence Jungo, Arnaud Kerhornou, Philippe Le Mercier, Damien Lieberherr, Patrick Masson, Anne Morgat, Salvo Paesano, Ivo Pedruzzi, Sandrine Pilbout, Lucille Pourcel, Sylvain Poux, Monica Pozzato, Manuela Pruess, Nicole Redaschi, Catherine Rivoire, Christian J. A. Sigrist, Karin Sonesson, Shyamala Sundaram and Anastasia Sveshnikova at the SIB Swiss Institute of Bioinformatics; Cathy H. Wu, Cecilia N. Arighi, Chuming Chen, Yongxing Chen, Hongzhan Huang, Kati Laiho, Minna Lehvaslaiho, Peter McGarvey, Darren A. Natale, Karen Ross, C. R. Vinayaka, Yuqi Wang and Jian Zhang at the Protein Information Resource.
